# Racial and Ethnic Differences in Risk and Protective Factors of Dementia and CIND in the United States

**DOI:** 10.1093/geroni/igaa057.2304

**Published:** 2020-12-16

**Authors:** Nasim Ferdows, Maria Aranda

**Affiliations:** 1 USC, Los Angeles, California, United States; 2 University of Southern California, Los Angeles, California, United States

## Abstract

Recent population-based studies have shown declines in dementia prevalence in high-income countries, suggesting that improved population cardiovascular health and rising levels of education in the past 25 year were associated with reduction of dementia risks. However, in the US, there are variations in educational attainment, prevalence and management of chronic diseases, and behaviors associated with poor cardiovascular health among racial and ethnic groups. We performed a retrospective analysis of 3,495 older individuals (65+) in 2016 who participated in Harmonized Cognitive Assessment Protocol (HCAP) subsample of the Health and Retirement Study (HRS), to examine racial/ethnic differences in risk and protective factors associated with dementia and cognitive impairment. Linking HCAP to HRS, we traced individuals back to 2000 and created a longitudinal data of HCAP population (2000-2016). We found that racial/ethnic differences in risk and in protective factors throughout the life-course were associated with racial and ethnic disparities in dementia prevalence.

